# Circulating tumor cells (CTCs) are associated with abnormalities in peripheral blood dendritic cells in patients with inflammatory breast cancer

**DOI:** 10.18632/oncotarget.10290

**Published:** 2016-06-25

**Authors:** Michal Mego, Hui Gao, Evan N. Cohen, Simone Anfossi, Antonio Giordano, Sanda Tin, Tamer M. Fouad, Ugo De Giorgi, Mario Giuliano, Wendy A. Woodward, Ricardo H. Alvarez, Vicente Valero, Naoto T. Ueno, Gabriel N. Hortobagyi, Massimo Cristofanilli, James M. Reuben

**Affiliations:** ^1^ Department of Hematopathology, The University of Texas MD Anderson Cancer Center, Houston, TX, USA; ^2^ Department of Breast Medical Oncology, The University of Texas MD Anderson Cancer Center, Houston, TX, USA; ^3^ Department of Radiation Oncology, The University of Texas MD Anderson Cancer Center, Houston, TX, USA; ^4^ Department of MD Anderson Morgan Welch Inflammatory Breast Cancer Research Program and Clinic, The University of Texas MD Anderson Cancer Center, Houston, TX, USA; ^5^ Currently at Department of Medical Oncology, Comenius University, School of Medicine, National Cancer Institute, Bratislava, Slovakia; ^6^ Currently at Department of Medical Oncology, The National Cancer Institute, Cairo University, Cairo, Egypt; ^7^ Currently at Medical Oncology, Istituto Scientifico Romagnolo per lo Studio e la Cura dei Tumori (IRST) – IRCCS, Meldola (FC), Italy; ^8^ Currently at Department of Clinical Medicine and Surgery, University Federico II, Naples, Italy; ^9^ Currently at Division of Hematology-Oncology at Northwestern University Feinberg School of Medicine, Northwestern University, Chicago, IL, USA; ^10^ Currently at Department of Medicine at Medical University of South Carolina, Charleston, SC, USA

**Keywords:** circulating tumors cells, innate immunity, adaptive immunity, dendritic cells, inflammatory breast cancer

## Abstract

CTCs are involved in tumor dissemination and are an independent prognostic factor in primary and metastatic breast cancer patients. Dendritic cells (DCs) are the most efficient antigen presenting cells and are comprised of plasmacytoid-(pDC) and myeloid-(mDC) derived DC subsets. This study aimed to correlate CTC counts with the peripheral blood DC immunophenotypes and functions of inflammatory breast cancer (IBC) patients. This study included 65 IBC patients. Peripheral blood (PB) was obtained from patients prior to starting a new line of chemotherapy for CTCs enumeration by CellSearch^®^ and DC phenotype and function by flow cytometry; the characteristics of DCs were then correlated with CTC counts and clinical outcome. Twenty-one (32.3%) patients with CTCs ≥5 had a significantly inferior overall survival (OS) compared to patients with <5 CTCs (p=0.045). In addition, patients with ≥5 CTCs had a lower percentage of mDCs capable of producing TNF-α before or after activation through the toll-like receptor (TLR), as well as a lower percentage of mDCs producing IL-12 after TLR-activation. There was a positive correlation between CTCs counts and expression of the activation (CCR7) and costimulatory (CD86) receptors on TLR-activated mDCs and pDCs, respectively. Moreover, presence of high percentage of mDC capable to produce increased levels of TNF-α was independently associated with inferior OS (p = 0.0006). An increase in the percentage of mDC producing TNF-α might induce a pro-inflammatory environment that could play a role in determining the poor clinical outcome in IBC patients and could add further prognostic value to CTCs.

## INTRODUCTION

Inflammatory breast cancer (IBC) is rare but the most aggressive form of breast cancer that accounts for about 2-5% of all breast cancers [1–3]. It is associated with poor prognosis, with a 10-year disease-free survival rate approaching 20-25% [4–9]. IBC is associated with early metastatic dissemination as suggested by higher numbers of circulating tumor cells (CTCs) compared to other forms of breast cancer [10].

CTCs represent a heterogeneous population of cancer cells that are involved in tumor dissemination and progression, and have been proven to be a prognostic factor in primary [11] and metastatic breast cancer (MBC) [12]. Peripheral blood (PB) represents an adversarial microenvironment for the survival of CTCs due to the presence of shear forces, immune cells, and anoikis. All these factors contribute to the inefficiency of the metastatic process [13–15]. Further, since innate and adaptive immune mechanisms are purportedly responsible for controlling tumor dissemination, minor perturbations of immune surveillance could favor a microenvironment conducive for the survival and dissemination of CTCs, ultimately leading to cancer progression [16].

Dendritic cells (DCs) are the most efficient antigen-presenting cells comprised of plasmacytoid- and myeloid-derived subsets that regulate host anti-tumor innate and adaptive immune responses, respectively. DCs signal through toll-like receptors (TLR) that recognize conserved molecular patterns on common pathogens. In preclinical studies, agonists of TLR7 and TLR8 (TLR7/8) have shown their ability to enhance antitumor responses via diverse mechanisms involving both maturation and activation of DCs [17, 18].

Activation induces expression of CCR5 (C-C chemokine receptor type 5) and CCR7 (C-C chemokine receptor type 7) chemokine receptors on the surface of DCs [19]. In addition, activation of DCs induced by TLR7/8 agonists results in increased secretion of interferon (IFN)-α, interleukin (IL)-12, and tumor necrosis factor (TNF)-α, as well as upregulation of costimulatory molecules such as CD80 and CD86, increased polarization towards Th1-type responses, and enhanced tumor lysis [20–22]. Specifically, plasmacytoid dendritic cells (pDCs) produce IFN-α that promotes immunogenic maturation of other DC populations or induces CD4+ T cells to differentiate into IL-10-producing T cells leading to immune tolerance [23] through expansion of T-regulatory (T-reg) cells [20]. On the other hand, impaired IFN-α production by pDCs could potentially affect the generation of effector CD8+ T cells and compromise antitumor responses [24, 25]. Moreover, production of IL-10 by pDCs may induce immune tolerance through the activation of T-reg cells [21]. Myeloid DCs (mDCs) regulate adaptive immunity through the production of IL-12 and TNF-α inducing maturation of DCs, macrophages and cytotoxic T cells [22].

Despite the well-known link between immune system function and cancer initiation and progression, current knowledge of the relationship between the immune system and CTCs in breast cancer is rather sparse. It was recently reported that MBC patients with ≥5 CTCs per 7.5 mL of PB had impaired natural killer (NK) cell function in comparison with that of patients with <5 CTCs [26]. Another study reported a differential expression of TLR on immune cells in MBC patients, based on the presence or absence of CTCs [27]. Specifically, patients with MBC who had CTCs ≥5 exhibited an increased percentage of DCs expressing TLR2, TLR4, and TLR8, and a lower percentage of DCs expressing TLR3, compared to patients with CTCs <5 [27]. As DCs are very efficient in presenting antigen to T cells, changes in the number or function of DCs are more likely to affect the ability of T cells in exerting an efficient cell-mediated immune response. Indeed, we recently reported abnormalities of the innate and adaptive immunity in IBC patients with ≥1 CTCs per 7.5 mL of PB, consisting in lower percentages of both CD3+ (pan-T) and CD3+/CD4+ (T-helper) cells, as well as NK cells, accompanied by a higher percentage of T-reg cells in PB [28].

In this translational study, we hypothesized that IBC patients with detectable CTCs in PB have lower numbers and compromised function of DCs responsible for poorer clinical outcome. Further, we aimed to correlate CTC numbers with the status and functions of DCs in PB of patients with IBC.

## RESULTS

### Patients’ characteristics

This translational study included 65 patients with IBC (21 stage III IBC, 14 *de novo* metastatic and 30 recurrent metastatic IBC) treated between October 2008 and April 2012 at the University of Texas MD Anderson Cancer Center. In the same cohort of patients we have previously reported association between T-cell immunity and CTCs [28]. The median age of the study subjects was 54 years (range, 33-76). Thirty-five (53.9%) patients were treatment naïve at the time of blood collection. Patients’ characteristics are shown in Table 1. The median baseline CTC count was 2 (range, 0-211) per 7.5 mL of PB. Among the 65 patients, 40 (61.5%) had CTC counts of ≥1 and 21 (32.3%) had CTC counts of ≥5. The proportion of patients with ≥1 CTC was lower in those with stage III disease compared to patients with metastatic IBC (mIBC) disease (33.3% vs 75.0%; *P* = 0.002). Consistently, fewer patients with stage III IBC had CTCs ≥5 than those with mIBC (5.0% vs 45.5%; *P* = 0.001). In univariate analysis, only disease stage was associated with CTC count, whereas there was no correlation of hormone receptor and HER2 status, tumor grade, and histological type with CTCs. Patients with Stage III IBC had a significantly lower percentage of monocytes and a higher percentage of mDC with TLR7/8-induced expression of CCR5 compared to patients with mIBC. Chemonaive patients had significantly higher white blood cell count, and dendritic cell count, and lower percentage of monocytes, mDC and mDC with TLR7/8-induced expression of CCR7 compared to chemotherapy pretreated patients (data not shown).

**Table 1:** Clinicopathological characteristics of patient tumors (n = 65)

	Stage III IBC		mIBC		*P*- value
	N	%	N	%	
**All**	21	100.0	44	100.0	NA
**CTC per 7.5 mL of blood**					
≥ 1	7	33.3	33	75.0	0.002
≥ 5	1	4.8	22	50.0	0.001
**Grade**					
1 and 2	9	42.9	14	31.8	0.58
3	12	57.1	29	65.9	
unknown	0	0.0	1	2.3	
**Histology**					
Invasive ductal carcinoma	18	85.7	36	81.8	0.74
Other	3	14.3	8	18.2	
**ER/PR status**					
Negative for both	6	28.6	24	54.5	0.07
Positive for either	15	71.4	20	45.5	
**HER2 status**					
Amplified	10	47.6	14	31.8	0.78
Normal	11	52.4	30	68.2	
**ER/PR and HER2/neu status**					
Triple receptor negative	5	23.8	17	38.6	0.28
Non-triple receptor negative	16	76.2	27	61.4	
**Sites of metastases**					
Non-visceral	NA	NA	27	61.4	NA
Visceral	NA	NA	17	38.6	
**Bone metastases**					
Present	NA	NA	23	52.3	NA
Absent	NA	NA	21	47.7	
**No. of metastasis**					
1	NA	NA	21	47.7	NA
≥2	NA	NA	23	52.3	
**Treatment**					
Treatment naïve	21	100.0	14	31.8	NA
Pretreated	0	0.0	30	68.2	

Abbreviations: NA, not applicable; ER, estrogen receptor; PR, progesterone receptor.

### Association between CTCs and percentage of DC subsets

Mean percentage (± standard error of mean – SEM) of total DCs among white blood cells for patients with <1 CTC was 0.3% ± 0.1% compared with 0.5% ± 0.1% for patients with ≥1 CTCs (*P* = 0.36). Similarly, there was no difference in the number of total DCs among patients with <5 CTCs vs with ≥5 CTCs (0.4% ± 0.1% vs. 0.5% ± 0.1%, *P* = 0.18). Moreover, there was no difference in the percentages of pDCs or mDCs between patients without CTCs *vs*. those with ≥1 CTC, or between patients with <5 CTCs *vs*. those with ≥ 5 CTCs (Table 2). Despite the lack of association between CTCs and percentages of pDCs and mDCs, we observed significant differences in the ability of mDCs to secrete cytokines, constitutively or in response to activation with TLR7/8 agonists, as reported below.

**Table 2:** Association between CTCs and percentage of different subpopulations of dendritic cells synthesizing cytokines

Variable	CTC < 1	CTC > 1	p – value	CTC < 5	CTC > 5	p - value
**Number of patients**	24.0	40.0	NA	43.0	21.0	NA
**WBC x 10^3^/uL**	6.6 ± 0.5	7.1 ± 0.4	0.24	6.8 ± 0.4	7.2 ± 0.5	0.45
**DC subsets**	0.3 ±0.1	0.5 ±0.1	0.36	0.4 ±0.1	0.5 ±0.1	0.18
**mDC in DC**	41.4 ±3.1	43.2 ±2.5	0.82	41.7 ±2.3	44.3 ±3.4	0.74
**pDC in DC**	18.1 ±2.1	21.4 ±1.7	0.51	21.2 ±1.6	17.8 ±2.4	0.12
**mDC_TNF_a.TLR**	59.1 ±4.5	52.7 ±3.5	0.43	60.5 ±3.1	43.5 ±4.6	**0.01**
**mDC_TNF_a.US**	1.9 ±1.5	2.7 ±1.1	0.29	1.9 ±3.4	1.1 ±1.6	**0.05**
**pDC_TNF_a.TLR**	73.6 ±4.9	63.0 ±3.8	0.08	71.1 ±3.6	58.3 ±5.3	0.38
**pDC_TNF_a.US**	2.1 ±2.3	2.9 ±1.8	0.48	1.6 ±1.7	4.8 ±2.5	0.88
**mDC_IFN_a.TLR**	0.05 ± 0.2	0.8 ±0.2	0.67	0.6 ±0.2	0.7 ±0.2	0.87
**mDC_IFN_a.US**	1.0 ±0.2	0.6 ±0.2	**0.03**	1.0 ±0.2	0.3 ±0.3	**0.05**
**pDC_IFN_a.TLR**	38.0 ±4.8	27.5 ±3.8	0.18	34.2 ±3.6	25.6 ±5.3	0.28
**pDC_IFN_a.US**	0.9 ±0.7	1.5 ±0.5	0.94	1.2 ±0.5	1.5 ±0.7	0.66
**mDC_IL_12.TLR**	25.9 ±4.1	20.2 ±3.1	0.22	26.5 ±2.9	13.7 ±4.1	**0.03**
**mDC_IL_12.US**	0.9 ±0.3	0.7 ±0.3	0.09	0.8 ±0.2	0.6 ±0.4	0.09
**pDC_IL_12.TLR**	4.3±1.6	4.7 ±1.2	0.32	3.8 ±1.1	6.2 ±1.6	0.64
**pDC_IL_12.US**	1.9 ±0.5	0.3 ±0.4	0.25	1.1 ±0.4	0.4 ±0.6	0.68
**mDC_IL_10.TLR**	16.5 ±3.1	16.4 ±2.4	0.79	17.1 ±2.3	15.0 ±3.4	0.31
**mDC_IL_10.US**	11.7 ±2.3	10.7 ±1.8	0.56	11.4 ±1.7	10.5 ±2.5	0.31
**pDC_IL_10.TLR**	5.6 ±1.5	6.2 ±1.1	0.98	5.5 ±1.1	7.0 ±1.6	0.93
**pDC_IL_10.US**	8.5 ±2.2	8.1 ±1.7	0.50	8.9 ±1.6	7.0 ±2.4	0.19

#### Association between CTCs and cytokine synthesis by DCs

DCs produce several cytokines that can either augment or suppress host immune function. A summary of the correlations found between CTCs and percentages of DC subsets, secreting cytokines constitutively or after activation with TLR7/8 agonists, is reported in Table 2. Patients with ≥1 CTC had a significantly lower percentage of mDCs constitutively secreting IFN-α comparing to patients with no CTCs (1.0% ± 0.2% vs. 0.6% ± 0.2%, P = 0.03). On the other hand, we observed significant differences in the percentage of mDCs that secreted cytokines constitutively, as well as in response to activation via TLR7/8 between patients with <5 CTCs opposite to patients with ≥5 CTCs (Table 2). Patients with CTCs <5 had a significantly higher percentage of mDCs that constitutively secreting TNF-α (1.9% ± 3.4%, vs. 1.1% ± 1.6%, P = 0.05) and IFN-α (1.0% ± 0.2%, vs. 0.3% ± 0.3%, P = 0.05) than that of patients with ≥5 CTCs. In addition, patients with CTCs <5 had a higher percentage of mDCs that synthesized TNF-α upon activation with TLR7/8 agonists than that of patients with ≥5 CTCs (60.5% ± 3.1% vs. 43.5% ± 4.6%, P = 0.01). Similarly, TLR7/8-activated mDCs showed higher percentages of IL-12-secreting cells in patients with CTCs <5 than in those with ≥5 (26.5% ± 2.9% vs. 13.7% ± 4.1%, P = 0.03). No difference in the percentage of mDCs was found between patients with <5 and those with ≥5 CTCs, irrespective of whether it was constitutive secretion of IL-10 or *de novo* synthesis of IL-10 following activation through TLR7/8.

#### Association between CTCs and DCs expressing chemokine receptors and co-stimulatory molecules

Next, DCs were evaluated for the constitutive and TLR7/8-induced expression of CCR5 and CCR7 chemokine receptors (activation/maturation markers), as well as CD80 and CD86 co-stimulatory molecules that enhanced their ability to activate T-cells. Table 3 lists a summary of association between CTCs and percentages of DC subsets expressing chemokine receptors and co-stimulatory molecules. There was no correlation between CTCs and either subset of dendritic cells using the threshold of 1, whereas significant correlation between CTCs and the expression of CCR7 (P = 0.04) and CD86 (P = 0.02) on TLR7/8-activated pDCs and mDCs, respectively, was found when considering the threshold of 5 CTCs.

**Table 3:** Association between CTCs and percentage of different subpopulations of dendritic cells expressing cytokine receptors and co-stimulatory molecules

Variable	CTC < 1	CTC > 1	p - value	CTC < 5	CTC > 5	p - value
**Number of patients**	24.0	40.0	NA	43.0	21.0	NA
**CCR5 in mDC**	55.8 ±5.8	63.4 ±4.7	0.25	61.6 ±4.5	57.8 ±6.5	0.64
**CCR5 in pDC**	75.1 ±7.0	73.3 ±5.7	0.55	76.5 ±5.3	68.7 ±7.8	0.64
**CCR7 in mDC**	9.6 ±2.5	3.2 ±2.0	0.46	7.1 ±1.9	2.7 ±2.8	0.21
**CCR7 in pDC**	2.8 ±1.8	1.9 ±1.4	0.19	2.5 ±1.3	1.6 ±2.0	0.09
**CCR5_in_mDC.TLR**	10.9 ±2.8	11.2 ±2.3	0.20	11.8 ±2.1	9.3 ±3.3	0.16
**CCR5_in_mDC.US**	21.8 ±3.5	18.2 ±2.9	0.15	20.0 ±2.6	18.8 ±4.1	0.95
**CCR5_in_pDC.TLR**	14.5 ±3.0	9.0 ±2.4	0.02	12.7 ±2.3	7.6 ±3.5	0.23
**CCR5_in_pDC.US**	22.3 ±4.2	14.6 ±3.5	0.18	18.6 ±3.2	15.5 ±5.0	0.62
**CCR7_in_mDC.TLR**	10.6±2.6	13.0 ±2.2	0.79	9.9 ±17.3	1.9 ±3.0	0.31
**CCR7_in_mDC.US**	6.1 ±1.8	6.2 ±1.5	0.76	6.1 ±1.4	6.3 ±2.1	0.75
**CCR7_in_pDC.TLR**	24.8 ±4.8	28.5 ±4.0	0.51	23.6 ±3.5	35.3 ±5.5	**0.04**
**CCR7_in_pDC.US**	5.3 ±2.2	4.2 ±1.8	0.72	5.7 ±1.6	2.1 ±2.6	0.23
**CD80_in_mDC.TLR**	65.8 ± 3.8	82.3 ±3.4	0.41	81.8 ±3.1	81.2 ±4.9	0.23
**CD80_in_mDC.US**	18.2 ± 3.1	21.9 ±2.6	0.65	19.0 ±2.3	23.7 ±3.7	0.82
**CD80_in_pDC.TLR**	65.8 ± 3.8	71.8 ±3.1	0.10	68.6 ±2.9	71.2 ±4.5	0.27
**CD80_in_pDC.US**	3.0 ± 1.3	2.5 ±1.1	0.55	2.4 ±1.0	3.4 ±1.5	0.96
**CD86_in_mDC.TLR**	92.6 ± 1.8	94.5 ±1.5	0.38	92.4 ±1.3	97.0 ±2.1	**0.02**
**CD86_in_mDC.US**	86.1 ± 2.8	93.7 ±2.3	0.07	89.2 ±2.2	94.0 ±3.4	0.16
**CD86_in_pDC.TLR**	91.5 ± 2.2	91.5 ±1.8	0.46	91.2 ±1.6	92.3 ±2.5	0.66
	35.3 ± 4.6	38.9 ±3.8	0.93	33.3 ±3.3	47.7 ±5.2	0.09

#### Multivariate analysis of association between CTCs and DCs subsets

In the multivariate analysis, only disease stage was associated with CTCs at the threshold of 5 (Table 4), whereas the percentage of mDCs synthesizing TNF-α after TLR7/8 activation was independently associated with the presence of ≥5 CTCs (Table 5).

**Table 4:** Multivariate logistic regression model for the binary indicator of CTC ≥ 1

Variable	Odds ratio	95% CI Low	95% CI Upper	*P* – value
**% mDC_IFN-α_US** (continous variable)	0.83	0.51	1.34	0.440
**Stage** mIBC vs. stage III	4.92	1.53	15.81	0.007

**Table 5:** Multivariate logistic regression model for the binary indicator of CTC ≥ 5

Variable	Odds ratio	95% CI Low	95% CI Upper	*P* – value
**% CD86_in_mDC_TLR** (continous variable)	1.11	0.98	1.26	0.112
**% mDC_TNF-α_TLR** (continous variable)	0.96	0.93	0.99	0.005

### Prognostic value of CTCs and DC subsets

At a median follow-up time of 19.4 months (range, 1.0-66.0 months), 42 patients (65.0%) had died. Patients with either ≥1 or ≥5 CTCs had a significantly inferior OS than patients with no CTCs or with CTCs <5, [HR=2.48, p = 0.003 and HR=1.85, p = 0.045, respectively] (Figure 1A). The levels of DC subsets were categorized as “low” or “high” based on the median percentage of DCs reported in the study population. Patients with high percentage of mDCs synthesizing TNF-α after TLR7/8 activation had inferior survival than those with low percentages of these cells (Table 6). In the multivariate analysis, CTCs, hormone receptor and HER2 status, and percentage of mDCs synthesizing TNF-α after TLR activation were associated with OS (Table 7).

**Table 6:** Prognostic value of different subpopulations of dendritic cells on overall survival in IBC patients

Variable	HR	95% Lower CI	95% Upper CI	p - value
**DC**	1.13	0.61	2.11	0.70
**mDC in DC**	0.63	0.34	1.17	0.14
**pDC in DC**	0.72	0.39	1.34	0.30
**CCR5 in mDC**	0.75	0.4	1.39	0.36
**CCR5 in pDC**	1.02	0.55	1.9	0.95
**CCR7 in mDC**	1.06	0.57	1.97	0.85
**CCR7 in pDC**	1.26	0.66	2.4	0.51
**mDC_TNF-α.TLR**	0.41	0.22	0.77	**0.004**
**mDC_TNF-α.US**	0.63	0.34	1.16	0.14
**pDC_TNF-α.TLR**	0.82	0.44	1.51	0.51
**pDC_TNF-α.US**	1.91	1	3.63	0.08
**mDC_IFN-α.TLR**	0.72	0.39	1.33	0.28
**mDC_IFN-α.US**	1	0.54	1.85	0.99
**pDC_IFN-α.TLR**	0.79	0.43	1.47	0.45
**pDC_IFN-α.US**	1.2	0.62	2.34	0.60
**mDC_IL-12.TLR**	0.92	0.49	1.72	0.79
**mDC_IL-12.US**	0.6	0.32	1.13	0.11
**pDC_IL-12.TLR**	0.64	0.34	1.2	0.16
**pDC_IL-12.US**	1.06	0.54	2.07	0.87
**mDC_IL-10.TLR**	1.22	0.66	2.25	0.52
**mDC_IL-10.US**	1.29	0.7	2.38	0.42
**pDC_IL-10.TLR**	1.1	0.59	2.03	0.77
**pDC_IL-10.US**	1.4	0.76	2.6	0.27
**CCR5_in_mDC.TLR**	1.29	0.69	2.42	0.42
**CCR5_in_mDC.US**	1.54	0.82	2.9	0.17
**CCR5_in_pDC.TLR**	1.67	0.89	3.14	0.11
**CCR5_in_pDC.US**	1.35	0.72	2.55	0.34
**CCR7_in_mDC.TLR**	0.84	0.45	1.57	0.58
**CCR7_in_mDC.US**	1.19	0.63	2.23	0.59
**CCR7_in_pDC.TLR**	1.4	0.74	2.63	0.29
**CCR7_in_pDC.US**	0.91	0.48	1.71	0.77
**CD80_in_mDC.TLR**	1.04	0.56	1.96	0.89
**CD80_in_mDC.US**	0.66	0.35	1.24	0.17
**CD80_in_pDC.TLR**	0.72	0.38	1.34	0.29
**CD80_in_pDC.US**	0.59	0.3	1.16	0.09
**CD86_in_mDC.TLR**	0.98	0.52	1.83	0.95
**CD86_in_mDC.US**	0.66	0.35	1.25	0.20
**CD86_in_pDC.TLR**	0.66	0.35	1.25	0.19
**CD86_in_pDC.US**	0.88	0.47	1.65	0.69

* all immune cells subpopulation were dichotomized as “low” or “high” based on median value of percentage of immunes cells in all patients. All hazard ratios are based on comparison of “low” vs. “high” groups.

**Table 7:** Multivariate analysis of prognostic factors associated with overall survival

Variable	HR (95% C.I.)	*P* - value
**Baseline CTCs count** ≥ 5 vs. < 5	3.451 (1.705 - 6.982)	0.0006
**HER2 status** Overexpressed vs. negative	0.372 (0.180 - 0.769)	0.0076
**Hormone receptor status** Positive for either vs. negative for both	0.213 (0.103 - 0.439)	< 0.00001
**mDC_TNF-α_TLR** High vs. low	3.114 (1.627 - 5.961)	0.0006
**pDC_TNF-α_US** High vs. low	0.499 (0.236 - 1.057)	0.0696

* all immune cells subpopulation were dichotomized as “low” or “high” based on median value of percentage of immunes cells in all patients.

**Figure 1A:**
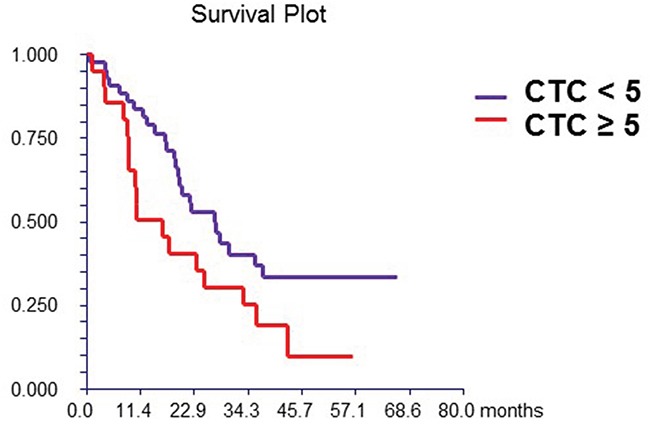
Prognostic value of CTC Patients with ≥ 5 CTCs/7.5mL of peripheral blood had had significantly better survival compared to patients with < 5 CTCs (hazard ratio [HR] = 1.85, 95% CI 0.94 – 3.67, p = 0.045)

Finally, we combined the prognostic values of CTCs and DCs. Patients with <5 CTCs and a low percentage of mDCs synthesizing TNF-α after TLR7/8 activation had significantly better outcome compared with that of patients with either ≥5 CTCs or high percentages of DCs, or with both these features (HR = 3.11, 95% CI 1.63 – 5.96, p = 0.0006) (Figure 1B).

**Figure 1B:**
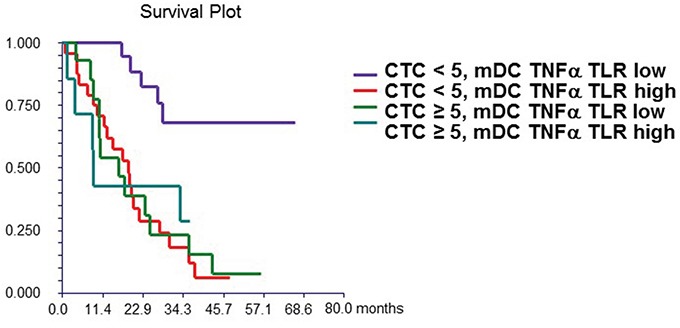
Combined prognostic value of CTC and mDC synthesizing TNF-α after TLR stimulation Patients with “high” mDC synthesizing TNF-α after TLR stimulation and CTC ≥ 5 (**group 4**) had significantly worse survival (median OS = 8.9 months) compared to patients with “low” mDC synthesizing TNF-α after TLR stimulation and CTC < 5 (**group 1**) (median OS not reached). Patients with either “high” mDC synthesizing TNF-α after TLR stimulation and CTC < 5 (**group 2**) (median OS = 18.6 months) or “low” mDC synthesizing TNF-α after TLR stimulation and CTC ≥ 5 (**group 3**) (median OS = 16.1 months) had intermediate prognosis. mDC synthesizing TNF-α after TLR stimulation were dichotomized as “low” or “high” based on median value of percentage of these cells in all patients.

## DISCUSSION

In a recent study, we reported on the prognostic value of CTCs in patients with IBC [10]. In this study, we observed that IBC patients with ≥1 CTCs or ≥5 CTCs per 7.5mL of peripheral blood have alterations in status and functions of peripheral blood DCs compared with IBC patients with no CTC or <5 CTCs, respectively. Although we did not observe differences in the absolute numbers of pDCs or mDCs according to CTC counts patients with ≥5 CTCs had a lower percentage of mDCs capable of *de novo* synthesis of TNF-α after TLR7/8 activation. In addition, we found that an increased percentage of mDCs synthesizing TNF-α after TLR7/8 activation was an independent prognostic factor for OS. DCs are an indispensable link between innate and adaptive immunity; importantly, several cancers exhibit dysfunction of DCs. Evidence from a recent study reporting increased expression of TLR on DCs of patients with MBC in the presence of detectable CTCs might reflect the occurrence of immune response to endogenous ligands and/or the existence of aberrant immune cells that are potentially involved in creating a cancer promoting microenvironment [Green, 2014]. We hypothesize that the observed inverse association between mDCs capable of *de novo* synthesis of TNF-α after TLR7/8 activation and OS could be related to high production of TNF-α favoring a tumorigenic microenvironment. It has been reported that the presence of myeloid-derived cells in tumor microenvironment [42] and the secretion of inflammatory cytokines by activated immune cells [43, 44] are associated with disease progression.

The activation of mDCs through TLRs or by inflammatory cytokines converts immature DCs into mature DCs that present specific antigen to T cells, thereby activating them [45]. Maturation of DCs is accompanied by upregulation of MHC class II and co-stimulatory molecules such as CD80 and CD86, and secretion of inflammatory cytokines such as IFN-α, IL-12, and TNF-α, IL-1 and IL-6 [46]. We observed positive correlations between ≥5 CTCs and the expression of CD86 on TLR7/8-activated mDCs, suggesting enhanced maturation and activation of DCs in patients with elevated CTC numbers.

Moreover, in the current study patients with ≥5 CTCs exhibited significant impairment in the ability of TLR-activated mDCs to secrete TNF-α and IL-12 (Table 2), potentially leading to an absent or reduced Th1-like response, similar to that observed in tumor microenvironment with myeloid-derived cells [42]. On the other hand, our data show that in patients with <5 CTCs, who are known to have a good prognosis [12], the concomitance of low levels of TNF-α secreted by mDCs is associated with a remarkably good prognosis (Figure 1B).

Increasing data suggest that tumor infiltrating immune cells in different types of cancer, including breast cancer are of prognostic value. Infiltration of tumor with different subtypes of immune cells including dendritic cells is closely associated with prognosis and/or response to anticancer treatment [47–49]. It has been shown that the infiltration of pDCs into the tumor microenvironment is associated with poor outcome in early breast cancer patients [50]. As pDCs are the principal producers of IFN-α [51] to initiate innate immune responses, suboptimal IFN-α production promotes the expansion of FOXP3(+) regulatory T cells, leading to poor prognosis in breast cancer patients [52]. In our study, we observed the prognostic value of the percentage of mDCs capable of synthesizing TNF-α after TLR activation and more importantly, we achieved increased prognostic value of CTCs in combination with this subtype of DCs (Figure 1). These data suggest that in addition to CTCs, peripheral blood DCs and their functional capabilities that can be easily measured are also biomarkers of prognosis in breast cancer.

It is known that epithelial-to-mesenchymal transition (EMT) plays an important role in tumor invasion and generation of CTCs. Our recent work suggests that the activation of immune cells in the tumor microenvironment by tumor cells leads to the secretion of soluble factors such as TNF-α by the activated immune cells and induction of the more aggressive EMT phenotype by the tumor [53]. Emerging data suggest CTCs are comprised of subsets that have undergone partial or complete EMT [54–56]. EMT is closely related to immunity and activation of the EMT program induces functionally impaired dendritic cells and T-regulatory cells, suggesting an a potential immunosuppressive effect of the EMT process. This is consistent with the association between CTCs and several subtypes of DCs [57], observed in our study. Thus, we suggest that high numbers of CTCs might be the consequence of a defective immune response including DCs. Moreover, the induction of EMT in CTCs might be associated with a tumor microenvironment that leads to defects in DCs (number and function) as well as an increase in CTCs counts.

The limitations of our study include the small sample size and patient heterogeneity. In this study, we combined data of stage III and IV IBC patients to increase statistical power of analyses; however, we always included disease stage as a covariate in the multivariate analyses to control for this confounder. Our results are applicable only to IBC patients and do not wish to imply that these results are generalizable to non-IBC patients. Further, approximately half of the patients were pretreated with chemotherapy that could have affected study results as well, especially due to decreases in monocyte counts associated with prior chemotherapy. Therefore, we included the percentage of cells and not the absolute cell count in our data analysis. To assess any relationship between CTCs and immune cells, we performed multiple comparisons suggesting that our results should be confirmed in further studies as they are only hypothesis generating, despite their biological and clinical rationale.

In conclusion, in this study, we showed for the first time that IBC patients with ≥ 5 CTCs had low percentages and impaired function in both subtypes of DCs. Together with previously observed abnormalities in T cells and NK cells, these data suggest general dysfunction of immune system in patients with inflammatory breast cancer who have an increased number of CTCs. Moreover, immune cell profiling could add further prognostic value to CTCs in IBC patients.

## PATIENTS AND METHODS

### Study patients

The Institutional Review Board (IRB) of The University of Texas MD Anderson Cancer Center approved this study (protocol LAB08-0199; Chair: J. Reuben). Patients with locally advanced or metastatic IBC, treated from October 2008 through April 2012, who provided written informed consent, were eligible. Patients underwent systemic therapy as appropriate for their malignancies, irrespective of the presence of CTCs or CTC count. Patients who suffered in the previous 5 years from a concurrent malignancy other than non-melanoma skin cancer were excluded.

All patients underwent pretreatment diagnostic biopsy. The diagnosis of IBC was based on clinical signs such as diffuse erythema, *peau d'orange*, tenderness, induration, and warmth [29, 30]. Clinical stage at diagnosis was coded according to the criteria set forth in the sixth edition of the American Joint Committee on Cancer's *AJCC Cancer Staging Manual* [31].

Data regarding tumor histologic subtype, hormone receptor status, human epidermal growth factor receptor 2 (HER2) amplification status, type and number of metastatic sites, and type of systemic therapy were recorded in all the patients and associated with the presence and number of CTCs and immune cells.

### Peripheral blood for *in vitro* studies

Atraumatic venous blood sampling was performed at the antecubital fossa. Before starting a new line of chemotherapy each patient provided 34.5 mL of peripheral blood of which 7.5 mL were used for CTC enumeration and the remaining 27 mL for immune assessment.

### Detection of CTCs in peripheral blood

The CellSearch system (Veridex Corporation, Warren, NJ) was used to detect and enumerate CTCs in 7.5 mL of peripheral blood, as previously described [32]. Specimens were stored at room temperature and analyzed within 1 day from phlebotomy.

### Quantification of circulating DCs

Subsets of DCs, including mDCs and pDCs were detected and enumerated in peripheral blood samples, as described previously [33–35].

### Cytokine synthesis by mDCs and pDCs activated through TLR

Cytokine synthesis by DCs after stimulation with 10 μM of CL097 (water-soluble derivative of the imidazoquinoline compound R848; Invivogen, San Diego, CA) was performed, as described previously [34, 35]. Similarly, CL097, TLR7 and TLR8 agonists (InvivoGen, San Diego, CA), was used to induce cytokine synthesis in DCs, through the activation of the NF-κB [36, 37].

### Statistical analysis

Patient characteristics were summarized using the median (range) for continuous variables and frequency (percentage) for categorical variables. Normality of distribution was tested by the Kolmogorov-Smirnov test. If data were normally distributed, sample means were tested by Student *t*-test or analysis of variance. For non-normally distributed data, the nonparametric Mann-Whitney *U* test or Kruskal-Wallis *H* test was used. Pearson's or Spearman's correlation was used according to the normality of the data.

Baseline CTC count was defined as the closest CTC measurement obtained before the beginning of a new line of systemic therapy. Baseline CTC counts were dichotomized using two different thresholds: as <1 *vs* ≥1 and as <5 *vs* ≥5 per 7.5 mL of PB. The cut-off of 1 CTC was chosen because it has been investigated in different settings, such as in primary breast cancer, including locally advanced and inflammatory breast cancer [10, 11, 38–41]. The cut-off of 5 CTCs was shown to be prognostic for progression-free survival (PFS) and overall survival (OS) in patients with MBC and in those with IBC [10, 12, 41].

Univariate analyses with the Chi squared or the Fisher's exact tests were performed to assess the association between immune cells and CTC status. Subsequently, a multivariate logistic regression analysis, including the variables significantly associated with baseline CTC counts in univariate analysis, was completed. A backward model selection was conducted, and the final fitted model is shown in Tables 4 and 5.

We correlated baseline CTC counts and percentages of DC subsets with OS. For survival analysis, percentages of all DC subsets were dichotomized as “low” or “high” category using the median count of all study patients. Median follow-up period was calculated as a median observation time among all patients and among those still alive at the time of their last follow-up visit. OS was calculated from the date of baseline CTC enumeration to the date of death or last follow-up visit. OS was estimated using the Kaplan-Meier product-limit method and compared between groups using the log-rank test. A multivariate Cox proportional hazards model for OS was used to assess differences in outcome according to baseline CTC counts, immune cells, hormone receptor status, HER-2 status, and tumor grade and stage. Step-wise regression techniques were used to build multivariate models using a significance level of 0.10 to remain in the model. All statistical tests were 2-sided, and *P* values <0.05 were considered statistically significant. Statistical analyses were performed using NCSS 2007 software (Hintze J, 2007, Kaysville, Utah, USA).
